# Exploring the impact of Crohn’s disease on the intragastric environment of fasted adults

**DOI:** 10.5599/admet.846

**Published:** 2020-06-15

**Authors:** Maria Vertzoni, Christina Koulouri, Androniki Poulou, Konstantinos Goumas, Christos Reppas

**Affiliations:** 1 Department of Pharmacy, National and Kapodistrian University of Athens, Zografou, Greece; 2 Department of Gastroenterology, Red Cross Hospital of Athens, Athens, Greece

**Keywords:** intragastric volume, pH, osmolality, surface tension, pepsin activity, bile salts

## Abstract

We explored the potential impact of Crohn’s disease on the intragastric environment of fasted adults with a view to potential effects on intragastric performance of orally administered drugs in the fasted state. Data were collected from 15 healthy individuals and 15 patients with Crohn’s disease. All subjects remained fasted for at least 12h prior to gastroscopy. Intragastric resting volume and pH were measured upon aspiration. Osmolality, surface tension, pepsin activity, and content of six bile acids were measured within 4 months upon sample collection. Unlike intragastric volumes, intragastric osmolality was significantly increased by Crohn’s disease. However, mean osmolality value in patients was only slightly higher than in healthy individuals (293 vs. 257 mOsmol/kg, respectively), therefore, unlikely to affect intragastric drug product performance. Primarily due to the high variability of data in healthy individuals, the potential effects on intragastric pH and surface activity could not be evaluated on a statistical basis. However, based on average (mean and median) values, even if they are statistically significant, it seems unlikely to be of clinical significance. Inter-subject variability of pepsin activity, and total bile acids content was high in both the healthy and the patients’ groups. Statistical investigation of the potential impact of Crohn’s disease on these parameters requires prior designation of the minimum differences to be detected; such differences will determine the minimum sample size required of relevant investigations.

## Introduction

During the last two decades, substantial progress has been made in characterizing physicochemically the gastrointestinal (GI) contents of healthy adults [1] resulting to the development of novel in vitro and in silico methodologies for evaluating the luminal fate of orally administered drug products [2]. However, relevant luminal data in patients with a GI disease are very limited [3].

Crohn’s disease is a heterogeneous disorder with a multifactorial etiology, including genetic factors, environmental insults and intestinal microbiota, characterized by chronic, segmental and transmural inflammation that affects the gastrointestinal tract and may involve any segment of the oral cavity up to the anus. The involvement of the upper GI lumen is less known, as a routine upper endoscopy is not commonly indicated [4].

To date, apart from few data on the pH along the GI lumen of patients with inflammatory bowel disease [5], data on the physicochemical characteristics of luminal contents have not been investigated [2]. Gastric contents, in addition to gastric acid, contain a variety of components that are either secreted by the stomach (e.g. enzymes, electrolytes, mucus), swallowed/ingested (e.g. saliva and, in the fed state, food components) or refluxed from the duodenum in the stomach (e.g. bile components) which can affect oral dosage form performance [1, 6].

The purpose of the present exploratory study was to evaluate whether the fasting gastric environment of patients could be affected by the Crohn’s disease.

## Experimental

### Collection and treatment of aspirates

Human aspirates were collected at the Red Cross Hospital of Athens, after receiving approval by the Scientific and the Executive Committee of the Hospital (AP 152/160307).

Fifteen healthy adults (21-60 years old, 8 males, 7 females) and fifteen adults with Crohn’s disease (18-60 years old, 8 males and 7 females) participated in the study during a routine gastroscopic procedure for diagnostic purposes at the Gastroenterology Department of Red Cross Hospital of Athens (Table 1).

Diagnosis of Crohn’s disease was based on histologic examination. Fourteen had the disease in the distal and one in the proximal region of the digestive tract. All subjects gave their informed consent for performing gastroscopy and collecting gastric contents.

**Figure 1.**
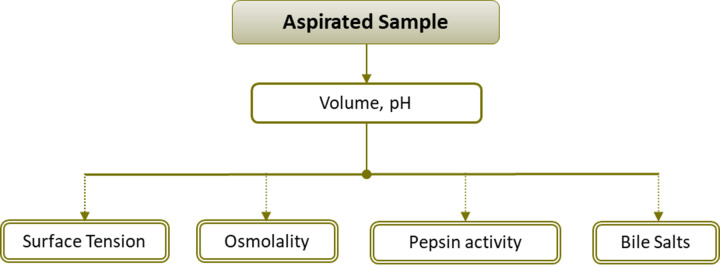
Measurements performed in samples aspirated in this study. A double lined box implies a specific vial that was stored at -70 °C prior to the relevant measurement.

Subjects remained fasted for at least 12 h prior to gastroscopy. Following the standard protocol, gastrointestinal fluids were aspirated during the procedure to facilitate the diagnostic process i.e., improve visibility of the gastric wall. Normally, these fluids are directed to the waste upon aspiration. Complete aspiration of gastric contents was performed to determine volume of resting gastric fluid. Upon aspiration, each sample was kept for 2 h in the freezer, its pH was measured, and it was then transferred to -70 °C. In all cases, to avoid multiple thaw-freezing cycles, prior to freezing the material to be stored was distributed to five smaller vials (Figure 1). Osmolality, surface tension, pepsin activity and content of six bile acids were measured within 4 months upon sample collection.

### Materials

Pepsin from porcine gastric mucosa was from Sigma Chemical Co., St. Louis, MO, USA. All other chemicals used were of analytical grade and all solvents were of LC-MS grade and were purchased from Sigma Aldrich Chemie GmbH.

### Analysis of Samples

pH values were measured by a pH electrode (Schott, modelCG842, Mainz, Germany). Surface tension was measured at 37 °C using the DeNouy ring method (Sigma 70 tensiometer, KSV Instruments LTD, Helsinki, Finland). Osmolality was measured by using the freezing point depression technique (semimicro osmometer Typ Dig L; Knauer, Berlin, Germany). Pepsin activity was measured by a modification of the method described by Anson, and quantification was based on pepsin from porcine gastric as a standard [7, 8]. The quantification limit was calculated to be 424 USP units/mL. Individual bile salts, that is taurocholates (TC), glycocholates (GC), taurochenodeoxycholates (TCDC), ursodeoxycholates (UDC), glycochenodeoxycholates (GCDC) and glycochenocholates (GDC)] were analyzed using HPLC-CAD as described previously [9].

### Data treatment

Raw data are presented as Box-Whisker plots showing the median, 10^th^, 25^th^, 75^th^, and 90^th^ percentiles and the individual outlying data points. Within each box, horizontal solid lines indicate median values and horizontal dotted lines indicate mean values.

Where possible, i.e. the power of the test allowed and normality and equal variance tests passed, the difference between the two groups was evaluated with the unpaired t-test. Type I error was set to 0.05. All statistical comparisons were performed using SigmaStat 3.5 (SPSS Science Inc., New York, USA).

## Results and discussion

**Figure 2.**
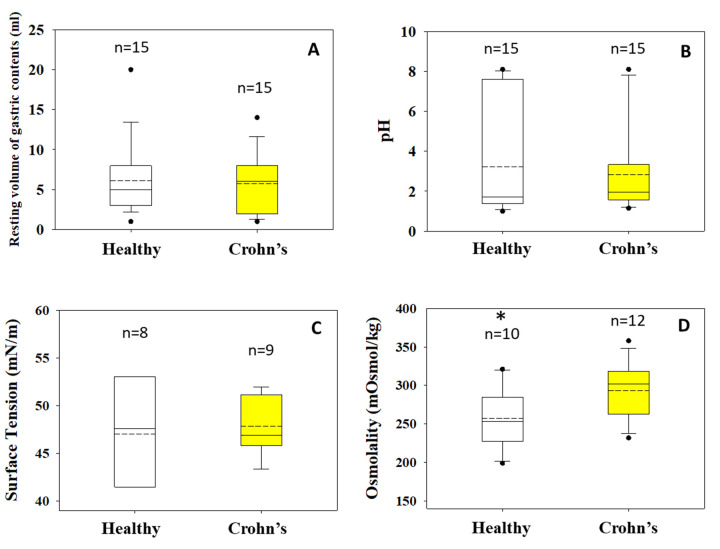
Resting volume of gastric contents (A), pH (B), surface tension (C) and pepsin activity (D) of the contents of stomach of healthy adults (white boxes) and Crohn’s disease patients (yellow boxes) measured in the fasted state. n is the number of subjects contributed to the construction of box plots. Asterisk (*) indicates that the difference is significant.

Mean (SD) values for the resting gastric volumes were 5.8 (3.6) mL and 6.1 (4.5) mL for Crohn’s disease patients and healthy subjects, respectively (Figure 2A). These values are smaller than the resting gastric fluid volumes reported previously, based on data collected with a water-sensitive magnetic resonance imaging technique, i.e. individual volumes ranged from 13 mL to 72 mL [10].

Intragastric pH values of patients with Crohn’s disease were less variable than in healthy adults. Median (range) pH values of gastric contents in Crohn’s patients and healthy adults were 2.0 (range: 1.2-8.1) and 1.7 (range: 1.0-8.1), respectively (Figure 2B). Similar pH values were observed in the literature. Median intragastric pH values estimated from data collected in 12 patients with Crohn’s disease and in 12 healthy adults using a radiotelemetry capsule were 2 and 1.55, respectively [11]. Interestingly, the difference was significant whereas no differences between patients in remission and with active disease were observed [11]. In contrast, using a free-floating pH-sensitive telemetric capsule, Ewe et al. observed no significant differences in intragastric pH values between Crohn’s disease patients with the disease active at the ileocecal regions (n=15) and healthy adults (n=15) [12].

Surface tension of gastric contents was less variable in Crohn’s disease patients (Figure 2C). Median (range) values in the Crohn’s disease patients and in healthy adults were 46.9 (43.4-52.0) and 47.6 (37.0-54.1), respectively.

Mean(SD) osmolality values of gastric contents were 293 (35) mOsmol/kg and 257 (39) mOsmol/kg in the Crohn’s disease and healthy adults, respectively (Figure 2D). Intragastric osmolality was slightly but significantly different in adults with Crohn’s disease (p= 0.034).

High inter-subject pepsin activity was observed in both the Crohn’s disease patients and the healthy individuals groups. Coefficients of variation were 101% and 94%, respectively. Median (range) pepsin activity values of Crohn’s disease gastric contents were 131,300 (range: < 424 - 595,300) USP units/mL and 137,000 (range: < 424 - 333,600) USP units/mL for the Crohn’s disease and healthy adults, respectively. Generally, these data are in agreement with a study in which the effect of malnutrition and subsequent re-feeding on digestive function in Crohn’s disease patients was investigated [13]. Gastric acid secretion was stimulated by a one-hour infusion of pentagastrin and continuous aspiration through the gastric port enabled measurement of gastric acid secretion. The malnourished Crohn’s patients (BMI <17 kg/m2) had a mean basal acid output of 0.64 ± 0.33 mEq/h compared with 3.85 ± 0.93 mEq/h in healthy adults (p< 0.01). Following the period of nutritional support, the basal acid output reached levels of 2.12 ± 0.88 mEq/h, not statistically different to the levels observed in healthy adults (p=0.06). In our study, Crohn’s patients were within the normal weight apart from one patient who had the disease for more than 14 years and his BMI was borderline to be underweight. It should be noted that a statistically significant decrease of BMI in Crohn’s disease patient compared to healthy adults was observed [mean (SD) BMI 22.0 (3.0) vs 25.5 (3.7), p= 0.009] (Table 1).

Individual bile acid concentrations were highly variable in both study groups; concentrations were below the quantification limit (6 μM) in 11 out of 15 patients and in 7 out of 15 healthy individuals. Coefficients of variation of individual bile salt concentrations in Crohn’s patients and in healthy individuals ranged between 290% and 387% and between 206% and 304%, respectively. The predominant bile salts were glycoconjugates and the relative mean luminal concentrations were GC > GCDC > GDC ∼TC ∼ TCDC > UDC (Table 2).

**Figure 3.**
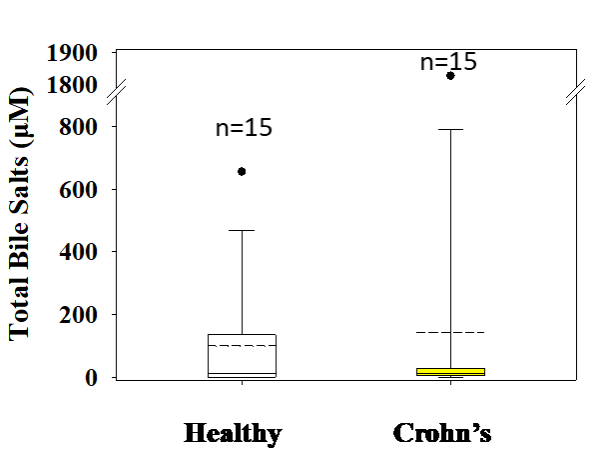
Total bile salts concentration of gastric contents of healthy adults (white boxes) and Crohn’s disease patients (yellow boxes) measured in the fasted state. n is the number of subjects contributed to the construction of box plots.

Mean (median) values for total bile salts concentration in the gastric contents of patients with Crohn’s disease and healthy individuals were 141(12) μΜ and 99(10) μΜ, respectively (Figure 3).

Based on pepsin activity data and bile acids data, investigation of the impact of potential differences between Crohn’s patients and healthy individuals requires prior designation of the minimum differences to be detected, as these will determine the minimum number of samples sizes required for reaching statistically relevant conclusions.

## Conclusions

Compared to healthy individuals, intragastric osmolality was significantly different but only slightly higher in Crohn’s patients; this difference is unlikely to affect the performance of drugs administered orally in the fasted state.

Based on this exploratory study, average values of all other characteristics also suggest that differences on the physicochemical characteristics of gastric contents between adults with Crohn’s disease and healthy adults if any, are unlikely to affect the performance of orally administered drugs in the fasted state.

However, especially for the most highly variable parameters, i.e. pepsin activity and bile acids concentrations, designation of the minimum differences to be detected is required in order to reach statistically relevant conclusions.

## Figures and Tables

**Table 1.** Demographics of individuals participated in the present investigation.

	Healthy adults			Adults with Crohn’s disease			
	Gender	Age (y)	BMI (kg/m^2^)	Gender	Age (y)	BMI (kg/m^2^)	Duration (y)
	F	26	21.19	F	18	19.05	< 0.5
	M	50	31.14	F	26	19.38	1
	M	34	22.22	M	40	24.31	0.5
	M	50	25.17	M	33	19.05	6
	F	52	23.44	F	27	19.53	10
	M	21	22.84	F	48	23.44	1
	F	58	26.22	F	52	25.71	Not confirmed
	M	60	29.41	M	60	23.96	1
	M	38	30.45	F	25	20.76	0.5
	F	38	31.02	M	35	29.22	4
	M	52	26.85	M	31	18.38	14
	F	25	19.53	M	19	21.37	1
	M	49	25.91	F	29	23.63	Not confirmed
	F	59	24.52	M	18	21.45	< 0.5
	F	59	22.04	M	19	21.14	Not confirmed
**Mean (SD)**		**44(13)**	**25.5(3.7)**		**32(13)**	**22.0(3.0)**	
**Median (min-max)**		**50** **(21-60)**	**25.2** **(19.5-31.1)**		**29** **(18-60)**	**21.4** **(18.4-29.2)**	

**Table 2.** Total and individual bile salts content of gastric contents of healthy and Crohn’s disease adults. In all cases, normality test failed; data are median (range); significance of difference between the two groups was tested with Mann-Whitney test; differences were not significant.

	Healthy	Crohn’s disease
Total bile salts (μM)	10 (0 - 656)	12 (0 - 1828)
Glycocholates	8 (<1 - 327)	7 (<1 - 490)
Glycochenodeoxycholates	<2 (<2 - 718)	<2 (<2 - 227)
Taurocholates	1 (<1 - 107)	<1 (<1 - 277)
Taurochenodeoxycholates	0 (0 - 81)	0 (0 - 342)
Glycodeoxycholates	<2 (<2 - 49)	<2 (<2 - 45)
Ursodeoxycholates	<1 (<1 - 10)	<1 (<1 - 15)
